# Hydrochlorothiazide as an Underrecognized Cause of Acute Pancreatitis: A Case Report and Literature Review

**DOI:** 10.7759/cureus.84633

**Published:** 2025-05-22

**Authors:** Daniel Hahn, Jasmine Fung, Angela Tran, Miruthu Varshini Jayaprakash Narayanan, George N Freg

**Affiliations:** 1 Internal Medicine, Touro College of Osteopathic Medicine, New York, USA; 2 Internal Medicine, St. Mary's General Hospital, Passaic, USA

**Keywords:** acute pancreatitis, drug-induced pancreatitis, hydrochlorothiazide, management of pancreatitis, pancreatitis

## Abstract

Acute pancreatitis is a common inflammatory disorder of the pancreas. While commonly caused by gallstones, alcohol use, and idiopathic causes, drug-induced acute pancreatitis (DIAP) is an often overlooked etiology that demands careful medication review and clinical vigilance. This case report describes a 72-year-old male patient with a history of hypertension and coronary artery disease who presented with severe epigastric pain radiating to the back, elevated lipase levels, and imaging findings consistent with acute pancreatitis. After excluding common causes such as gallstones, alcohol use, and hypertriglyceridemia, hydrochlorothiazide (HCTZ) was identified as the likely trigger. Discontinuation of hydrochlorothiazide led to clinical improvement and resolution of laboratory abnormalities. This case highlights the importance of considering drug-induced acute pancreatitis, particularly in patients with comorbidities who present with unexplained acute pancreatitis due to their pharmacological therapy.

## Introduction

Acute pancreatitis is a common gastrointestinal disorder and the leading cause of gastrointestinal-related hospitalization in the United States [1]. It is characterized by the sudden inflammation of the pancreas, which can range from mild and self-limited disease to severe forms associated with significant morbidity and mortality. Acute pancreatitis can be classified into three categories based on severity, according to the Revised Atlanta Classification: mild (no organ failure or complications), moderately severe (transient organ failure or local complications), and severe (persistent organ failure) [2]. The mortality rate ranges from approximately 3% in patients with mild edematous pancreatitis to up to 20% in those with pancreatic necrosis [3].

Clinically, acute pancreatitis typically presents with acute, severe epigastric abdominal pain that often radiates to the back and is often accompanied by nausea and vomiting. On physical examination, patients may exhibit epigastric tenderness, guarding, a low-grade fever, and, in severe cases, signs of a systemic inflammatory response. The best initial diagnostic step includes measuring serum lipase and amylase levels, with lipase being more specific and typically elevated to more than three times the upper limit of normal. Imaging, particularly contrast-enhanced computed tomography (CT), is the best confirmatory test for visualizing pancreatic inflammation, ruling out necrosis or peripancreatic fluid collections, and identifying alternative abdominal pathologies. Drug-induced acute pancreatitis (DIAP), on the other hand, is considered a diagnosis of exclusion. Common causes of acute pancreatitis must be ruled out initially, such as gallstones and chronic alcohol usage. DIAP is then confirmed with observed improvement in symptomatology after discontinuation of the drug in suspicion.

While the majority of cases are linked to gallstones or alcohol use, less prevalent causes include hypertriglyceridemia and adverse drug reactions [4]. Although rare, DIAP accounts for less than 5% of all cases and is especially important in recurrent or idiopathic cases [5]. Several drugs have been implicated in drug-induced acute pancreatitis, such as azathioprine, didanosine, valproic acid, and tetracyclines. Hydrochlorothiazide (HCTZ), a commonly prescribed thiazide diuretic for hypertension and fluid retention, has been reported in isolated cases as a potential cause of drug-induced acute pancreatitis. Hydrochlorothiazide's mechanism of action works by inhibiting sodium reabsorption in the distal convoluted tubules, promoting diuresis, and reducing blood pressure. Common side effects include hypotension and electrolyte imbalances, such as hypokalemia, hyponatremia, hyperuricemia, and hypercalcemia.

This case report highlights an instance of hydrochlorothiazide-induced acute pancreatitis in a 72-year-old patient who presented with a chief complaint of epigastric pain and hypertensive urgency. A comprehensive workup, including laboratory tests and imaging, revealed that the recent reinitiation of hydrochlorothiazide had instigated the episode of acute pancreatitis, and discontinuation of the drug led to clinical improvement, supporting a diagnosis of hydrochlorothiazide-induced acute pancreatitis. This case highlights the need for a high level of suspicion for medication-induced pancreatitis, especially in patients presenting with atypical symptoms or recent changes in their pharmacological treatment. As of late, there are 18 published papers on HCTZ-induced DIAP, underscoring the rarity of this condition and its clinical significance. This case report reinforces the importance of clinicians recognizing DIAP as a potential, albeit rare, side effect of HCTZ use, notably in patients with a supportive clinical presentation.

## Case presentation

The patient is a 72-year-old man with a past medical history of hypertension and coronary artery disease with a percutaneous stent placed 11 years before the current presentation. He presented to the emergency department with a chief complaint of hypertensive emergency associated with chest and abdominal pain.

The patient stated that the day before presenting to the emergency room, he checked his blood pressure thrice, averaging 210/120. Based on outpatient records, his average baseline blood pressure reading was 151/86. From his primary care physician, the patient was prescribed aspirin 81 mg daily orally, metoprolol succinate ER 25 mg daily orally, rosuvastatin 10 mg daily orally, ergocalciferol 1,250 mcg daily orally, and a combination pill of valsartan 80 mg and hydrochlorothiazide 12.5 mg daily orally. The patient has been prescribed these medications for the past six years; however, the patient states that he has been noncompliant with his hypertensive medications and takes them as needed. The day before presenting to the emergency department, because of the elevated blood pressure readings, the patient stated that he started to take his combination pill of valsartan 80 mg and hydrochlorothiazide 12.5 mg.

The day of the presentation, he stated that initially, there was a sharp subxiphoid chest pain and epigastric abdominal pain that radiated to his back toward his scapular area. The pain had a burning sensation and was associated with nausea and multiple episodes of non-bloody vomiting. The pain had a sudden onset and was rated eight out of 10. He denied experiencing shortness of breath, fever, recent illnesses, palpitations, lower extremity edema, dizziness, or episodes of syncope. The patient denied a history of smoking cigarettes or drinking alcohol.

The patient appeared in moderate distress. His physical examination revealed a regular rate and rhythm on the cardiac examination and clear lungs on bilateral auscultation during the pulmonary examination. The abdomen was soft, non-tender, and non-distended, with no guarding or rebound tenderness on abdominal examination. Due to a history of chronic hypertension and radiating chest pain, the patient was initially evaluated for possible myocardial infarction or aortic dissection. In the emergency department, the patient was found to have a moderately elevated troponin level (13.7 pg/mL), but this was within the patient's baseline. The electrocardiogram showed normal sinus rhythm without ST-segment or T-wave changes. The echocardiogram showed no wall abnormalities. Computed tomography angiogram of the thorax and abdomen with contrast showed no signs of aortic dissection.

**Figure 1:**
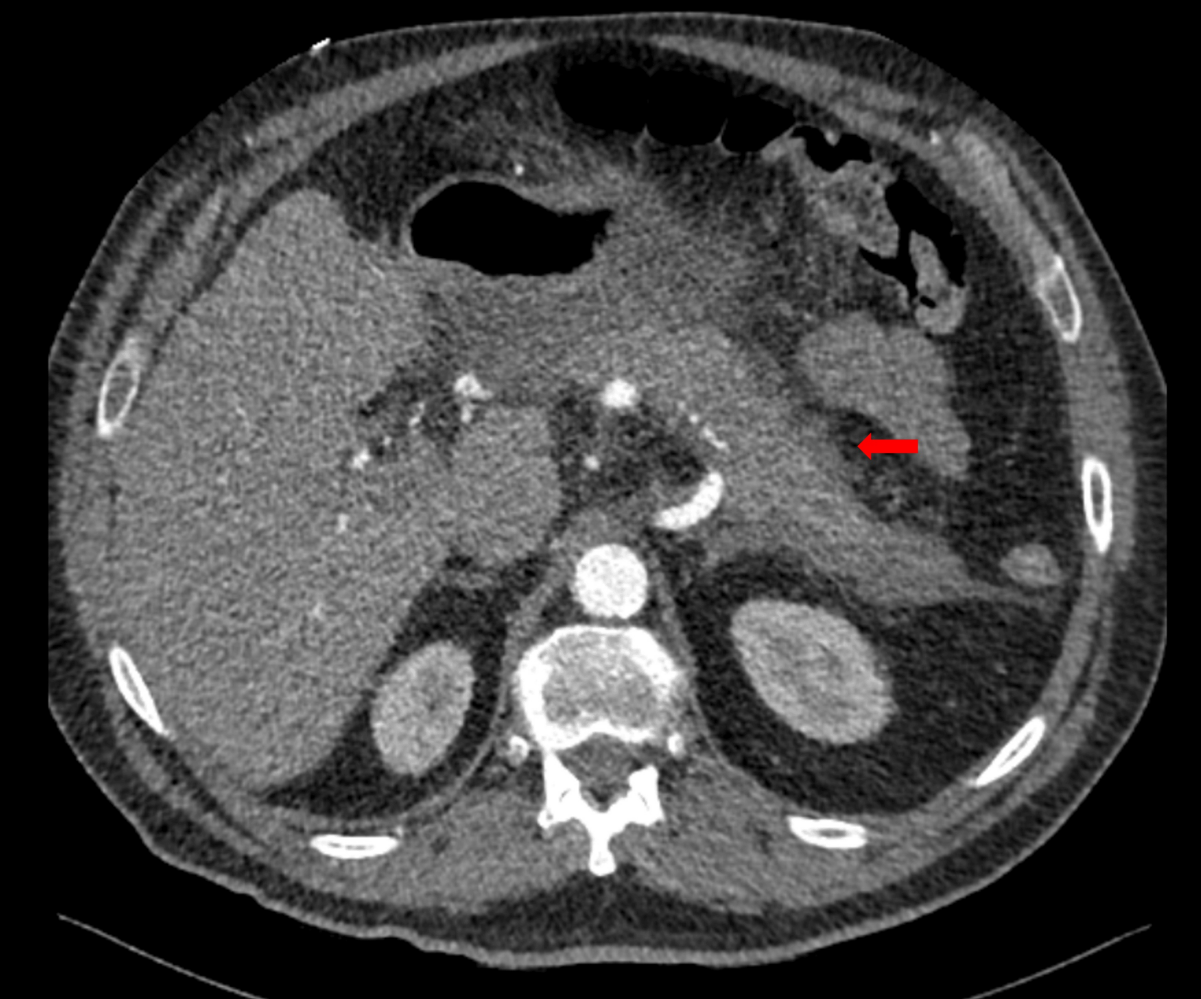
Computed tomography angiogram of the abdomen showing a diffusely enlarged pancreas Red arrow: diffusely enlarged pancreas with significant abnormal density in the peripancreatic region

However, during the initial laboratory workup, the patient was found to have elevated lipase levels of 1,471 units per liter (U/L). Furthermore, computed tomography revealed that the pancreas was diffusely enlarged, and there was significant abnormal density in the peripancreatic region (Figure 1). Additionally, no focal mass lesion or pseudocyst formation was seen within the pancreas. There are multiple small gallstones observed within the gallbladder, but no frank biliary ductal dilatation was evident. Minor pleural effusion was also present. These findings correlated with a diagnosis of acute pancreatitis.

**Figure 2:**
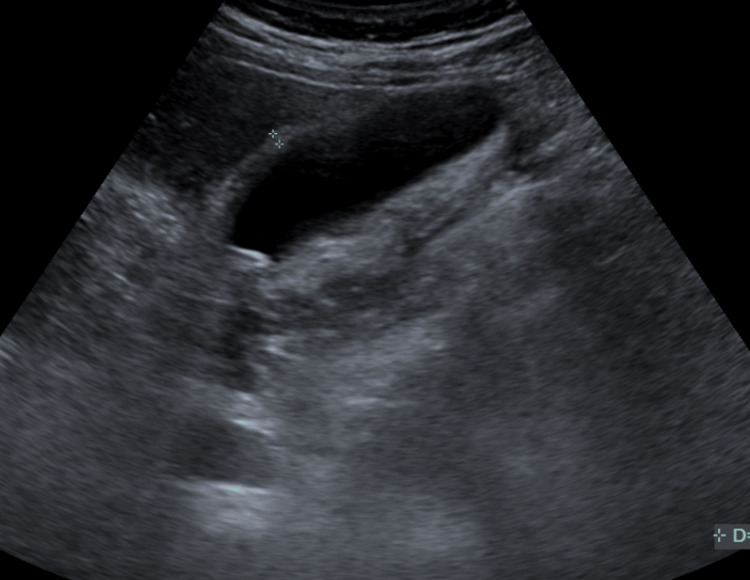
Ultrasound of the gallbladder and common bile duct with no common bile duct dilatation

A subsequent abdominal ultrasound was conducted, which showed that the gallbladder was fluid-filled and contained multiple small calculi. There was no gallbladder wall thickening. The common bile duct measured 4.00 mm and showed no common duct dilatation (Figure 2). These findings ruled out gallstone pancreatitis and added additional evidence toward acute pancreatitis.

**Figure 3:**
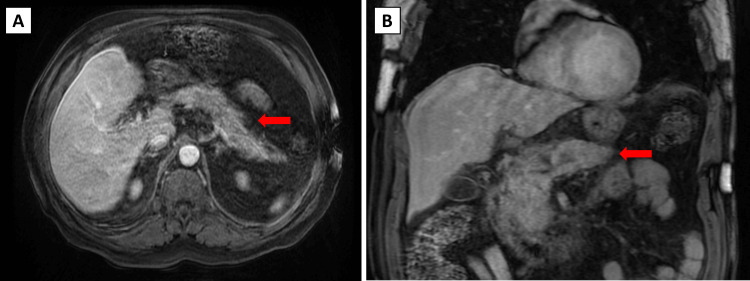
Magnetic resonance cholangiopancreatography showing enlargement of the pancreatic head and proximal body with surrounding peripancreatic fluid A: transverse section, B: coronal section Red arrows: diffusely enlarged pancreas with peripancreatic fluid

Lastly, a magnetic resonance cholangiopancreatography (MRCP) was conducted, which showed the gallbladder displaying a few small dependent stones. No pericholecystic fluid or wall thickening is seen. The common bile duct again was normal in caliber. There is no evidence of choledocholithiasis. However, the pancreas demonstrates an enlargement of the head and proximal body, with peripancreatic fluid consistent with acute pancreatitis (Figure 3A, 3B). There is no evidence of a pseudocyst or evidence of pancreatic necrosis.

Moreover, the patient's cholesterol levels were checked to evaluate for the potential for hyperlipidemia and hypertriglyceridemia, due to their association with gallstones. Although the levels were elevated, they were within the patient's baseline and not considered a cause for the acute pancreatitis (Table 1).

**Table 1:** Lipid panel laboratory values at initial presentation Cardiac risk: <5%, indicating "low risk," as defined by the 10-year risk of manifesting clinical cardiovascular disease (coronary artery disease, stroke, peripheral vascular disease, congestive heart failure, and cardiac death). It is calculated by patient age, sex, total cholesterol, HDL, systolic blood pressure, treatment for hypertension, smoking status, and diabetes status. HDL: high-density lipoprotein, LDL: low-density lipoprotein, VLDL: very-low-density lipoprotein

Lipid profile	Units	Reference range	Day 1, 13:37
Cardiac risk	%	-	3.3
Total cholesterol	mg/dL	<200	265
HDL	mg/dL	>60	79.7
LDL direct	mg/dL	<100	180
Triglycerides	mg/dL	<150	52
VLDL	mg/dL	5-30	10.4

Based on the laboratory results and imaging findings, a diagnosis of acute pancreatitis was made. The patient's Bedside Index for Severity in Acute Pancreatitis (BISAP) score was calculated at 2 points, due to his age and the presence of a pleural effusion, indicating a minimal risk of mortality from acute pancreatitis. The patient was started on aggressive hydration with 0.9% normal saline intravenous fluids. He was prescribed intravenous ketorolac 15 mg/mL every six hours for pain management and intravenous ondansetron 4 mg orally as needed to manage his nausea. The patient was placed on a nil per os (NPO) diet.

Additionally, the patient's hydrochlorothiazide was discontinued on day 1 at 16:23 PM. All other home medications, including valsartan, were continued during his hospitalization. Throughout the hospitalization, the patient's gastrointestinal-related laboratory values, including lipase, were monitored for clinical improvement and to track trends (Table 2). Total bilirubin and direct bilirubin levels remained normal, a pertinent negative for gallstone pancreatitis. Notably, the lipase levels decreased from 1,471 U/L (day 1 at 13:37), to 625 U/L (day 2 at 5:57), to 157 U/L (day 3 at 4:42). The only significant clinical management associated with these downward-trending levels of lipase was the removal of the patient's hydrochlorothiazide. Moreover, although valsartan has been associated with rare cases of pancreatitis, the patient continued to receive it, and it did not aggravate his pancreatitis.

**Table 2:** Laboratory values, including lipase and liver function tests, during hospitalization AST: aspartate aminotransferase, ALT: alanine transaminase

Test	Unit	Reference range	Day 1, 13:37 PM (emergency department)	Day 2, 05:57 AM (internal medicine department)	Day 3, 04:42 AM (internal medicine department)
AST	U/L	10-40	342	132	53
ALT	U/L	7-56	797	520	314
Alkaline phosphatase	U/L	44-147	112	91	88
Total protein	g/dL	6.0-8.3	7.2	6.7	6.3
Albumin	g/dL	3.4-5.4	4.0	3.7	3.5
Total bilirubin	mg/dL	0.1-1.2	0.7	0.9	1.0
Bilirubin, direct	md/dL	0.0-0.3	-	0.26	-
Lipase	U/L	0-160	1,471	>625	157
Calcium	mg/dL	8.6-10.2	10.3	9.8	9.6

Repeat troponin and creatine kinase tests were conducted and found to be within the patient's normal baseline values (Table 3).

**Table 3:** Cardiac profile laboratory values during hospitalization

Test	Unit	Reference range	Day 1, 11:33 AM (emergency department)	Day 2, 05:57 AM (internal medicine department)
Total creatine kinase	U/L	52-336 (male)	182	19.1
Troponin T	ng/L	<14	13.7	19.4

After a three-day hospital course, the patient was discharged with changes in his medication. Although the patient initially presented with a hypertensive urgency, discontinuation of the hydrochlorothiazide and resolution of the acute pancreatitis helped normalize his blood pressure values (Table 4).

**Table 4:** Blood pressure measurements before and during the hospitalization course BP: blood pressure, mmHg: millimeters of mercury

Date and time	Day 0 (at home)	Day 1, 07:00 AM	Day 1, 2:00 PM	Day 1, 07:00 PM	Day 2, 3:00 AM	Day 2, 07:00 AM	Day 2, 2:00 PM	Day 2, 07:00 PM	Day 3, 07:00 AM
Systolic BP (mmHg)	210	193	147	152	158	161	157	149	147
Diastolic BP (mmHg)	120	107	76	84	92	94	92	79	87

After discontinuing the hydrochlorothiazide, he was started on amlodipine 5 mg daily orally for hypertension management. He also switched from valsartan to losartan 50 mg daily orally. The patient was discharged with no additional gastrointestinal or pain complaints.

## Discussion

This clinical case presents a rare case of drug-induced pancreatitis with hydrochlorothiazide as the causative agent. Based on the Naranjo causality assessment, the patient had a score of 7, indicating a probable adverse drug reaction associated with hydrochlorothiazide. The most commonly observed causes of acute pancreatitis are gallstones, chronic alcohol usage, hyperlipidemia, endoscopic retrograde cholangiopancreatography (ERCP) trauma, and pancreatic tumors [6]. However, drug-induced acute pancreatitis, while a rare etiology, has been found in the literature [7]. Drug-induced acute pancreatitis is often overlooked because its onset is typically due to commonly used medications [8]. Thiazide diuretics are among the most frequently prescribed diuretics, accounting for 20%-25% of all medications used to treat hypertension in the United States, with 90%-95% being hydrochlorothiazide specifically [9]. However, pancreatitis is not one of this medication class's classically associated side effects [10].

When diagnosing acute pancreatitis, a patient must meet two of three criteria: abdominal pain, elevated serum lipase three times higher than the upper limit of normal, and confirmed findings on imaging [1]. Drug-induced pancreatitis is considered a diagnosis of exclusion, after ruling out common causes such as gallstones and chronic alcohol usage. It is then confirmed with observed improvement in symptomatology after discontinuation of the drug in suspicion [7].

With this diagnostic approach, it is imperative to understand the frequency of how often drug-induced pancreatitis is identified in the literature and the most common drugs implicated. The rate of drug-induced pancreatitis is 0.1%-2% of all acute pancreatitis incidents [11]. The most common drugs implicated in drug-induced pancreatitis are listed as class I medications, documented to have greater than 20 cases. The class I medications are didanosine, asparaginase, azathioprine, valproic acid, pentavalent antimonials, pentamidine, mercaptopurine, mesalamine, estrogen preparations, opiates, tetracycline, cytarabine, steroids, trimethoprim/sulfamethoxazole, sulfasalazine, furosemide, and sulindac. Class II medications are implicated in at least 10 cases of acute pancreatitis. They are rifampin, lamivudine, octreotide, carbamazepine, acetaminophen, phenformin, interferon alfa-2b, enalapril, hydrochlorothiazide, cisplatin, erythromycin, and cyclopenthiazide [12]. Because of this case-based classification system, physicians should report rare cases of acute pancreatitis caused by specific medications, such as hydrochlorothiazide.

The existing literature supports that multiple antihypertensive medications can cause acute pancreatitis, including angiotensin II receptor blockers, such as losartan, and beta-blockers, such as pindolol [6,13]. Among the reported cases, one confounding variable is the multidrug treatment plan in patients with chronic hypertension, making it difficult to isolate the sole causative agent [14]. Another case study raised the concern that hypertensive patients treated more aggressively with a combination drug regimen are more likely to develop pancreatitis than those who are prescribed a singular therapy [15]. Our patient, because of his chronic history of hypertension, was prescribed multiple medications, such as valsartan-hydrochlorothiazide, metoprolol, and rosuvastatin, adding to the concern of discerning one causative agent of pancreatitis or if there was a synergistic adverse effect of a multidrug treatment regimen. However, our patient stated multiple times that he was not compliant with his hypertensive medication and only started to take his valsartan-hydrochlorothiazide the day before presenting with severe abdominal and chest pain. Moreover, the withdrawal of the hydrochlorothiazide while continuing all his other home medications resulted in the resolution of the patient's acute pancreatitis, suggesting that hydrochlorothiazide was the key inciting factor.

Based on the literature, the possible mechanisms of drug-induced pancreatitis include cytotoxicity, metabolic reactions, and pancreatic duct constriction [16]. Multiple studies have cited hypercalcemia as a proposed mechanism for thiazide-induced pancreatitis [8,16]. However, although our patient experienced fluctuations in metabolic and electrolyte trends, no abnormal changes in calcium were observed. Instead, the patient's initial hyperkalemia, hyperglycemia, and elevated aspartate aminotransferase (AST) and alanine transaminase (ALT) levels underscore the systemic effects of acute pancreatitis compounded with volume depletion, concurrent hepatic stress, and disrupted endocrine function. A key clinical finding of this case is the chronic hypertension and acute hypertensive urgency of 210/120 mmHg. A hypertensive surge may indicate a systemic inflammatory response, possibly due to an adverse reaction to the hydrochlorothiazide or to the pancreatitis that ensued [17].

Reported cases of thiazide-induced pancreatitis highlight a commonality of older patients with concomitant underlying conditions [18]. In this case, a 72-year-old man with a past medical history of coronary artery disease and chronic hypertension suggests a predisposition to this adverse effect of thiazide usage. Despite his susceptibility, according to the Bedside Index for Severity in Acute Pancreatitis (BISAP), the patient's score was calculated to be 2 points, which is a lower-than-expected mortality from pancreatitis based on common etiologies. Instead, the risk was secondary to the adverse reaction of hydrochlorothiazide.

Differential diagnoses that must be considered when diagnosing acute pancreatitis may include alcohol-induced pancreatitis, cholelithiasis, or the presence of pancreatic lesions [19]. However, the patient had no history of alcohol usage. Moreover, the abdominal ultrasound and MRCP showed the absence of gallstones in the common bile duct, normal ductal diameter measurements on multiple imaging studies, and no lesions in the pancreas, ruling out these more common etiologic factors.

A limitation of the case was that it was not possible to directly measure the hydrochlorothiazide levels within the patient's serum based on the hospital's laboratory capabilities. Having quantifiable toxic levels would have added additional credence to the causality of the case presentation. Moreover, based on the Naranjo adverse drug reaction probability scale, a greater probability of causation could have been established if the patient had a previous instance of pancreatitis associated with hydrochlorothiazide use, hydrochlorothiazide was readministered and the same adverse reaction reappeared, or if a placebo was given and the pancreatitis did not reappear.

When treating drug-induced acute pancreatitis, it is essential to remove the suspected inciting agent, provide fluid resuscitation to maintain organ perfusion, manage pain, and offer nutritional support. A potential complication is necrotizing pancreatitis, which can result in a high mortality rate [15]. In these instances, necrotic tissue can become an infection, necessitating surgical intervention [20].

## Conclusions

This case highlights a rare but important case of a patient presenting with characteristic symptoms and laboratory findings of acute pancreatitis without a common etiology. The patient's elevated lipase levels and magnetic resonance cholangiopancreatography showing an enlarged pancreas were consistent with acute pancreatitis. However, imaging showed no ductal obstruction, ruling out gallstone-induced pancreatitis, and the patient had no history of alcohol use, ruling out alcohol-induced pancreatitis. However, with the discontinuation of hydrochlorothiazide, the patient's symptoms resolved. This case study reinforces the necessity of maintaining a high index of suspicion for drug-induced pancreatitis, especially in commonly prescribed medications such as hydrochlorothiazide. This case also serves as a reminder to clinicians to take caution in considering hydrochlorothiazide-induced pancreatitis in older patients with comorbidities, as their risk is heightened. It is a reminder that prompt removal of the offending agent leads to more favorable outcomes.
